# Improved Storage Properties and Cellular Uptake of Casticin-Loaded Nanoemulsions Stabilized by Whey Protein-Lactose Conjugate

**DOI:** 10.3390/foods10071640

**Published:** 2021-07-15

**Authors:** Bei Zhang, Miao Lei, Wenjing Huang, Gang Liu, Fangcheng Jiang, Dengfeng Peng, Qingrong Huang, Weiping Jin

**Affiliations:** 1 School of Food Science and Engineering, Wuhan Polytechnic University, Wuhan 430023, China; B15827049116@163.com (B.Z.); 18707194179@163.com (M.L.); huangwenjingbest@163.com (W.H.); lg820823@163.com (G.L.); fj109@scarletmail.rutgers.edu (F.J.); 2 Department of Food Science, Rutgers University, New Brunswick, NJ 08901, USA; 3 Key Laboratory of Oilseeds Processing, Ministry of Agriculture, Oil Crops Research Institute, Chinese Academy of Agricultural Science, Wuhan 430062, China; pengdengfeng@caas.cn

**Keywords:** casticin, nanoemulsion, whey protein isolated, conjugate, cellular uptake

## Abstract

Casticin has wide-ranging functional activities, but its water solubility is poor in food products. Here, a nanoemulsion stabilized by Maillard whey protein isolate conjugates (MWPI) was fabricated to encapsulate casticin. The nanoemulsion, with an average diameter of 200 nm, possessed the capability to load 700 μg/g casticin. MWPI-stabilized nanoemulsion showed better stability than that of the WPI nanoemulsion during 4 weeks of storage. Both the inhibition effects of the casticin-loaded nanoemulsion on cancer cells and the process of cellular uptake were studied. Results revealed that the casticin-loaded nanoemulsion had better inhibitory activity in HepG2 and MCF-7 cells than free casticin. Cellular uptake of the nanoemulsion displayed a time-dependent manner. After the nanoemulsion passed into HepG2 and MCF-7 cells, it would locate in the lysosome but not in the nucleus. The main pathway for the nanoemulsion to enter HepG2 cells was pinocytosis, whereas, it entered MCF-7 predominantly through the clathrin-mediated pit. This work implies that MWPI-stabilized nanoemulsions could be utilized as an effective delivery system to load casticin and have the potential to be applied in the food and pharmaceutical industries.

## 1. Introduction 

Casticin, 5,3′-dihydroxy-3,6,7,4-tetramthoxyflavone, is a kind of polymethoxy-flavonoid (PMF) extracted from the traditional Chinese medicine *Vitex* (*Fructus Vititcis*) [1]. Numerous studies have demonstrated that casticin has potential functional activities, including anti-inflammatory [2], anti-hyperprolactinemia [3], and anti-cancer [4]. Ramchandani et al. found that casticin significantly inhibited the proliferation of oral, lung, hepatocellular carcinoma, cervical, and bladder cancer cells. [5] One molecule of casticin contains four methoxy groups that are connected to the flavone ring. This structure possesses a strong planar structure that resists the entry of water [6]. The poor water-solubility of casticin decreases its bioavailability and limits its application in designing functional foods.

The fabrication of an emulsion is a potential way to encapsulate the poorly water-soluble PMFs and improve their bioavailability. Because hydrophobic bioactive compounds are easy to dissolve in the oil phase, they can be kinetically stable dispersed into the aqueous phase after forming nanoemulsions. Due to the small size (20–200 nm), nanoemulsions have the advantages of a large specific surface area, high stability, and adjustable rheology properties [7,8]. When the hydrophobic bioactive compounds are encapsulated in the nanoemulsion, they can be accurately delivered to a specific location in vivo, thereby enhancing their physical stability, reducing side effects, and improving bioavailability at the same time [9,10]. Many research papers have reported nanoemulsions as good delivery systems for improving the biological activity of PMFs [11,12]. Lei et al. fabricated a nanoemulsion to encapsulate nobiletin and immobilized it in an alginate hydrogel. Results showed that the nanoemulsion controlled the release of nobiletin and improved its stability during digestion and storage [13]. Soy protein isolate-stabilized nanoemulsions encapsulated the supersaturated tangeretin, and, with the addition of glycerin, the high loading of tangeretin was maintained and stabilized [14].

Glycosylate-modified protein, mainly through the Maillard reaction, possesses suitable amphiphilicity, which can provide steric hindrance to prevent the aggregation and disproportionation of emulsion droplets [15]. Mengíbar et al. evaluated the emulsifying ability of chitosan and β-lactoglobulin conjugates developed through the Maillard reaction, and the results showed that high-molecular-weight chitosan (56 and 39 kDa)-β-lactoglobulin conjugates had better emulsifying ability than unreacted protein [16]. Nagaraju et al. found that nanoemulsions stabilized by Maillard conjugates formed by sodium caseinate and polysaccharides (maltodextrin and pectin) had higher stability than that of nanoemulsions prepared by protein or polysaccharide separately [17]. WPI is commonly considered a high-quality protein supplement, though its stability is easily affected by temperature and pH [18]. Wang et al. fabricated WPI-inulin to improve the functional properties of WPI near its pI [19]. Xu et al. prepared a WPI-beet pectin conjugate-stabilized β-carotene emulsion and found it improved the centrifugal transmission profile and the freeze–thaw stability of the emulsion [20]. 

In the current work, the Maillard whey protein isolate-lactose conjugate (MWPI) was prepared and utilized as an emulsifier to improve the stability of the nanoemulsion. Casticin was loaded in the MWPI-stabilized nanoemulsion to inhibit the growth of MCF-7 and HepG2 cancer cells. Finally, the kinetics and mechanism of the cellular uptake of nanoemulsion were preliminarily clarified.

## 2. Material and Methods

### 2.1. Materials and Chemicals

The whey protein isolate (WPI) was gifted from Hilmar, USA. The casticin was extracted from *Vitex*, and the purity was 94.6% after comparison with a standard by HPLC. The casticin standard (≥98%, V117963), L-lactose, and dimethyl sulfoxide (biochemical grade, ≥99.7%) were purchased from Aladdin Reagent Co., Ltd. (Shanghai, China). The MCF-7 and HepG2 cell lines were obtained from the China Center for Type Culture Collection (CTCC, Wuhan, Hubei, China). Fetal bovine serum (FBS) and Dulbecco’s modified eagle’s medium (DMEM) for cell culture were purchased from Gibco (Grand Island, NE, USA). Penicillin-streptomycin and 0.25% trypsin were obtained from Jinuo Biomedical Technology Co., Ltd. (Hangzhou, China). Phosphate buffer saline (PBS) was purchased from Hyclone (Logan City, UT, USA). Cell-culture flasks with a capacity of 25 cm^2^, 96-well cell-culture plates, and cryotubes were purchased from Corning Co., Ltd. (Shanghai, China). Nile red and coumarin-6 were purchased from Aladdin Reagent Co., Ltd. (Shanghai, China). Lyso-Tracker red (a lysosomal red fluorescent probe) and 4,6-diamino-2-phenyl indole (DAPI) were purchased from Beyotime Biotechnology Co., Ltd. (Shanghai, China) and Solarbio Technology Co., Ltd. (Beijing, China), respectively.

### 2.2. Preparation and Characterization of WPI-Lactose Maillard Conjugate

#### 2.2.1. Preparation of WPI-Lactose Maillard Conjugate (MWPI)

WPI-lactose Maillard conjugate was fabricated according to the method reported by Liu et al. with some modifications [21,22]. Briefly, the powders of WPI and lactose were dissolved in DI water together with a ratio of 1:2 (*w/w*), and the final solid content was 5–10%. The mixture solution was adjusted to pH 7.0 and stirred for 12 h. After the solution was freeze-dried, the powders were placed in a desiccator with saturated KBr (relative humidity 79%). The WPI-lactose Maillard conjugates were obtained after reacting at 70 °C for 24 h. 

#### 2.2.2. Fourier Transform Infrared Spectroscopy (FTIR) Analysis

The powders of WPI and WPI-lactose Maillard conjugates were mixed with KBr separately, and then they were pressed into thin slices. The FTIR spectra of powders were measured with a NEXUS670 spectrometer (Nikolai Instruments Inc., Potwin, KS, USA). The wavenumber ranged from 4000 to 400 cm^−1^. The resolution and the number of scans were 4 cm^−1^ and 32, respectively.

#### 2.2.3. Sodium Dodecyl Sulfate Polyacrylamide Gel Electrophoresis (SDS-PAGE)

The sample (2 mg/mL) of WPI and MWPI were diluted 5 times with the loading buffer, centrifuged at 6000 rpm for 5 min, and then boiled for 5 min to complete denaturation. According to the protocol of the SDS-PAGE kit (Beyotime Biotechnology, Shanghai, China), the 12% separating gel and 5% concentrated gel were used for separation. Operation proceeded at 60 V for the first 30 min, and then changed to 120 V. Then, the gel was stained with 0.25% Coomassie Brilliant Blue R-250 for 30 min, and de-stained. An image was obtained using a Bio-Rad scanner (Hercules, CA, USA).

### 2.3. Preparation and Characterization of MWPI-Stabilized Nanoemulsions

#### 2.3.1. Preparation of the WPI/MWPI-Stabilized Nanoemulsion

The WPI or MWPI powder was dissolved in 10 mM PBS (pH 7.4) at a concentration of 1.0 wt%, and the oil phase fraction was set at 10%. The MWPI-stabilized nanoemulsion was fabricated via high-speed homogenization (Ultra-turrax T18IKA, GmbH, Berlin, Germany) at 10,000 rpm for 10 min, and a high-pressure homogenization (AH-2010, ATS Industrial Systems, GmbH, Berlin, Germany) with 900 Mbar and 10 cycles in succession.

#### 2.3.2. Storage Stability Test

Nanoemulsions stabilized by WPI and MWPI were placed at 4 °C, and 0.02% sodium azide was added in emulsions to prevent bacteria growth. The particle size of nanoemulsions measured by Zetasizer Nano-ZS (Malvern, UK) at 1 week, 2 weeks, and 4 weeks was recorded as the indicator for evaluating storage stability. 

### 2.4. Fabrication of Casticin-Loaded Nanoemulsions 

#### 2.4.1. The Solubility of Casticin in Oil

An excess of casticin was added to 10 g medium chain triglyceride (MCT), and magnetically stirred overnight at room temperature. After centrifugation at 5000× *g* for 15 min, 200 μL of the supernatant was mixed with 800 μL methanol and then filtered through a 0.45 μm filter for high performance liquid chromatography (HPLC) analysis. The samples (20 μL) were analyzed by a reverse-phase C18 column (150 μm × 4.6 μm, Shim-pack VP-ODS C18 column, Japan) with buffer A (ultrapure water) and buffer B (acetonitrile) at a flow rate of 1 mL/min. The detection wavelength was 254 nm. The gradient elution method was as follows: from 40% to 55% buffer B for 10 min, from 55% to 70% buffer B for 5 min, from 70% to 80% buffer B for 5 min, and held at 40% buffer B for 10 min.

#### 2.4.2. Preparation of Casticin-Loaded Nanoemulsion

The CAS was dissolved in MCT in a saturated state as an oil phase. The water phase was a 1.0 wt% MWPI solution. The homogenization process was carried out as described in Section 2.3.1. 

#### 2.4.3. Spray Drying of the Casticin-Loaded Nanoemulsion

The MWPI-stabilized nanoemulsion was spray dried under the following conditions: inlet air temperature 115 °C, feed rate 14 mL/min, spray airflow 1000 L/h, and needle frequency 1 time/15 s. Finally, the obtained powder was kept in a sealed bag protected from light, at 4 °C.

#### 2.4.4. Determination of Casticin Content in Nanoemulsion

The casticin in the nanoemulsion was extracted by adding 1 mL of n-hexane to 1 g of nanoemulsion, followed by vortexing and centrifugation at 10,000× *g* for 10 min. Next, the content of casticin was analyzed using HPLC as described in Section 2.4.1.

#### 2.4.5. Stability of Casticin-Loaded Nanoemulsion in the Culture Medium

The nanoemulsion was diluted 10-fold with DMEM (for cell culture, without FBS) and stored at 37 °C for 24 h. The particle size and polydispersity (PDI) of the nanoemulsion were recorded using a Marlven Nanosizer ZS90 (Marlven, UK).

### 2.5. Inhibitor Effects of Casticin-Loaded Nanoemulsion on Cancer Cells

MTT (3-(4,5-Dimethylthiazol-2-yl)-2,5-diphenyltetrazolium bromide) was used to evaluate the ability of the nanoemulsion to inhibit the proliferation of MCF-7 and HepG2 cells. Briefly, cells were seeded on 96-well plates in the exponential growth phase, and then the nanoemulsion was diluted with DMEM to control the final concentrations of casticin. After incubation, the nanoemulsion was removed, and each well was washed with PBS 2~3 times. Finally, 100 μL DMEM and 10 μL MTT solution were added to the wells and incubated for 4 h. Then, the medium was removed and 150 μL DMSO/well was added to dissolve the formazan crystals. The absorbance at 570 nm was recorded on a microplate reader (iMark, BIO-RAD, Hercules, CA, UAS). Untreated cells were defined as the negative control, and their OD value was recorded as 100% survival.

Inhibition rate = (1 − OD_sample_/OD_control_) × 100% (1)

### 2.6. Cellular Uptake of the Casticin-Loaded Nanoemulsion

First, Nile red was added in the MCT as the fluorescence dye before preparing the nanoemulsion. Then, 1 mL of cell suspension (2 × 10^4^ cells/mL) was inoculated into the CLSM dish and cultured for 24 h (37 °C and 5% CO_2_). After removing the medium and washing the cell surface with PBS, the casticin-loaded nanoemulsion labeled with Nile red was added and cultured for 10 min, 20 min, 30 min, 1 h, and 2 h. Before CLSM observation, the nanoemulsion was removed and the cells were washed three times by PBS buffer. The nuclei were stained with DAPI for 15 min.

### 2.7. Localization of Casticin-Loaded Nanoemulsion in Cells

Because lysosomes were dyed using LysoTracker red, the oil phase in the nanoemulsion was dyed with coumarin-6, which is a type of green fluorescence dye. The operation of the cell culture was similar to the description in Section 2.5. The coumarin 6-labeled nanoemulsion was added and incubated for 20 min. The cell surface was washed three times with PBS and fixed with 4% paraformaldehyde for 30 min. After washing with PBS three times, LysoTracker Red was used to label the lysosomes for 1 h. Before CLSM observation, cells were washed three times with PBS, and the nuclei were labeled with 100 μL DAPI for 15 min.

### 2.8. Cellular Uptake Mechanism of Casticin-Loaded Nanoemulsion

MCF-7 and HepG2 cells were inoculated into a CLSM dish as described in Section 2.5. To study the effect of temperature and inhibitors on the cellular uptake of the nanoemulsion, cells were pretreated at 4 °C or by adding 25 μg/mL dynasore, 10 μg/mL chlorpromazine, and 15 μg/mL amiloride HCl dihydrate for 30 min, respectively. Then, the coumarin 6-labeled nanoemulsion was incubated with the cells for 20 min. The surface of the cells was washed three times with PBS and observed under a CLSM.

### 2.9. Statistical Analysis

Each measurement of the samples was repeated at least three times, and the data was expressed as the average values and standard error in figures. The data were analyzed by SPSS 23 (SPSS Inc., Chicago, IL, USA) via one-way ANOVA. The significance level was *p* < 0.05, and figures were drawn with Origin 2017 (Origin Lab., Northampton, MA, USA).

## 3. Results and Discussion

### 3.1. Characterization of MWPI

**Figure 1:**
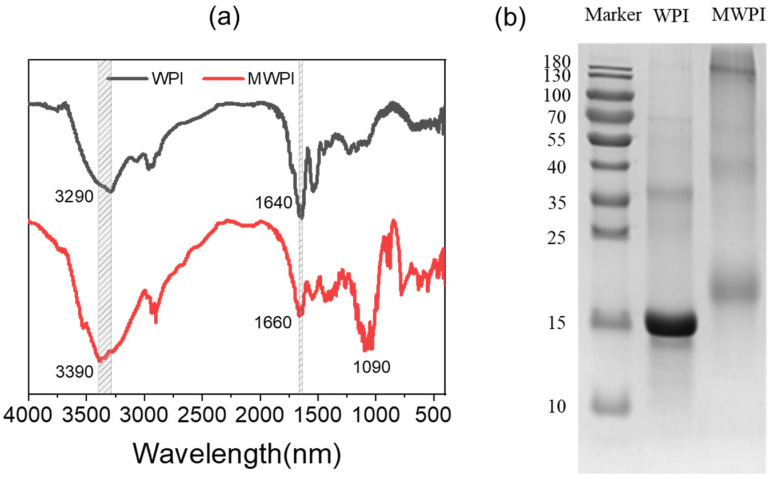
FTIR spectra (**a**) and SDS-PAGE (**b**) of WPI and MWPI.

The FTIR spectra of WPI and MWPI are shown in Figure 1a. The main characteristic peaks of WPI were 3290 cm^−1^ (N-H stretching and hydrogen bonding), 1640 cm^−1^ (amide I band, C=O stretching), 1540 cm^−1^ (amide II band, N-H bending), and 1390 cm^−1^ (amide III band, C-N expansion) [23,24]. As for MWPI, the absorption peak at 3290 cm^−1^ was shifted to 3390 cm^−1^, which might be ascribed to the stretching vibration of free-OH or the deformation vibration of the N-H bond. The peak at 1640 cm^−1^ is derived from the α-helix or random coil structure. After modification, the peak of WPI at 1640 cm^−1^ was shifted to 1660 cm^−1^, indicating that the α-helix or random coil structure had been changed. Compared with WPI, MWPI had a new absorption peak at 1000–1260 cm^−1^, which might be attributed to the stretching vibration of the C-O bond, resulting from the presence of multiple hydroxyl groups on the sugar chain. Figure 1b shows the SDS-PAGE of MWPI and WPI. The WPI mainly displayed a main band of 15 kDa and 35 kDa, which corresponded to β-lactoglobulin and dimers of β-lactoglobulin. After glycosylation, the molecular weight of the protein increased to 19 kDa and 40 kDa. It implied that WPI was successfully combined with lactose through Maillard reaction. 

### 3.2. Storage Stability of WPI and MWPI-Stabilized Nanoemulsions

**Figure 2:**
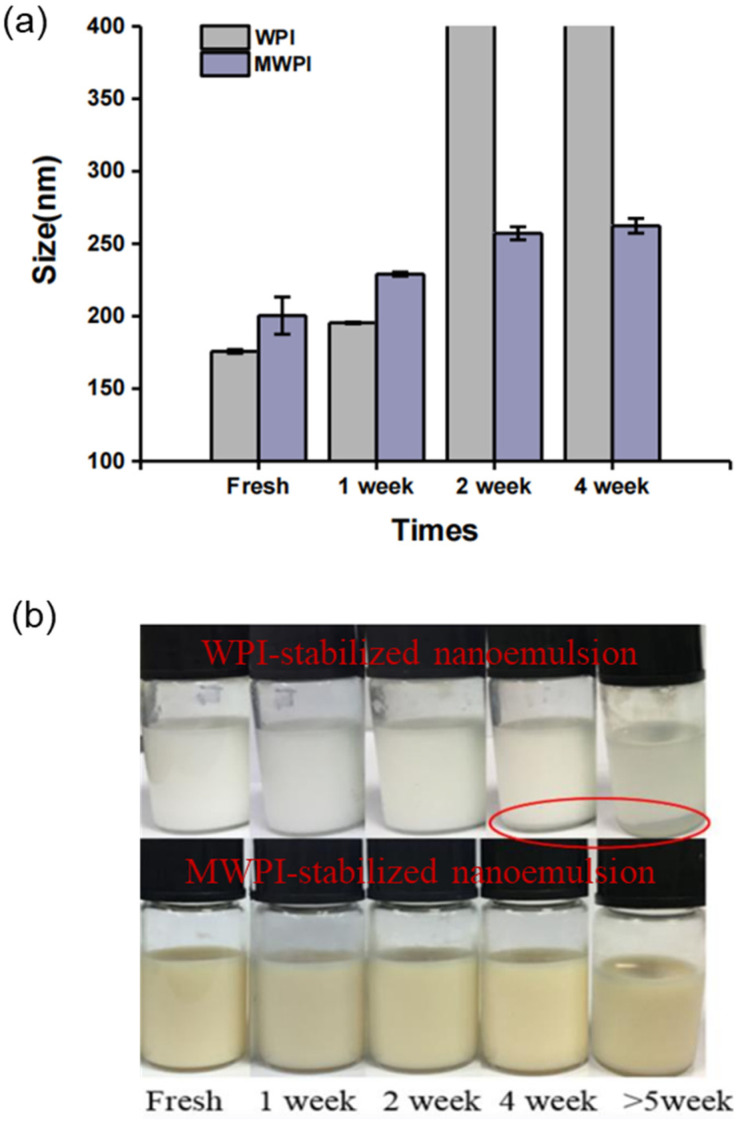
The particle size diagram (**a**) and appearance diagram (**b**) of nanoemulsions prepared with WPI and MWPI as emulsifiers over time.

Figure 2a shows changes of particle size in nanoemulsion stabilized by WPI and MWPI during the storage. The particle size of the freshly prepared WPI and MWPI nanoemulsions was 180 nm and 200 nm, respectively. As the storage time increased, the particle size of the WPI-stabilized nanoemulsion increased significantly. However, the particle size of the nanoemulsion stabilized by MWPI remained at around 250 nm after storage for 4 weeks. The accordant changes in the visual appearance of the nanoemulsions were clearly observed (Figure 2b). In the 4th week, the WPI-stabilized nanoemulsion began to cream, while the MWPI-stabilized nanoemulsion did not obviously change. This implied that MWPI had better emulsion storage stability than WPI. The Maillard reaction is a non-enzymatic reaction that forms covalent bonds between proteins and polysaccharides. The hydrophobic groups of proteins are anchored on the oil droplet surfaces, while the hydrophilic chains of polysaccharides generate a strong steric repulsion between oil droplets.

### 3.3. Characteristics of Casticin-Loaded Nanoemulsion

The solubility of casticin in MCT was 2.05 mM. MCT is a medium-chain fatty acid with low viscosity, high transparency, and good compatibility and is widely applied in the pharmaceutical, cosmetic, and food industries. The casticin-saturated MCT and whey protein isolate-lactose conjugate were chosen to fabricate the nanoemulsion via high-speed homogenization and high-pressure homogenization, and then the nanoemulsion was spray-dried. According to HPLC analysis, the casticin content in the spray-dried casticin-loaded nanoemulsion powder was 700 μg/g. 

**Figure 3:**
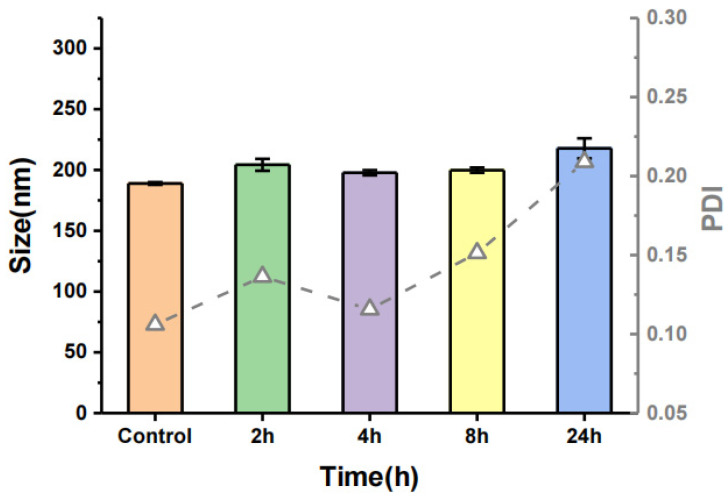
Particle size and PDI of CAS-NEs in the cell culture medium (DMEM) within 24 h; the particle size is represented by bars and PDI by lines.

The stability of the casticin-loaded nanoemulsion in DMEM medium was characterized by evaluating the particle size. Figure 3 shows the particle size of the casticin-loaded nanoemulsion after adding it into DMEM. At the beginning of the reaction, the particle size of the nanoparticles was about 200 nm, and there was no significant change from the blank group, indicating that casticin did not change the particle size of the casticin-loaded nanoemulsion. Over 24 h, the particle size of the casticin-loaded nanoemulsion did not change significantly, and the PDI was also less than 0.3, indicating that the emulsion had good stability and uniform dispersion. In summary, the casticin-loaded nanoemulsion maintained an unchanged particle size and PDI over 24 h.

### 3.4. Cell Anticancer Activity of the Casticin-Loaded Nanoemulsion

**Figure 4:**
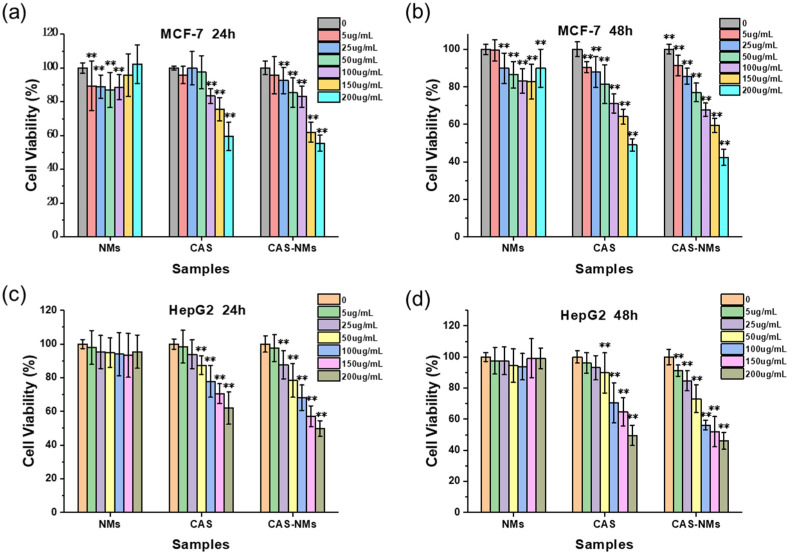
Cell viability of MCF-7 (**a**,**b**) and HepG2 (**c**,**d**) incubated with blank nanoemulsions (NMs), free casticin (CAS), and CAS-loaded nanoemulsions (CAS-NMs) for 24 h and 48 h respectively, which ** means *p* < 0.01 as compared to the control group.

Because liver cancer and breast cancer are among the cancers with the highest incidence in China and in women in the world, respectively, the typical cell lines of Hep G2 and MCF-7 cell lines of the two were chosen as models. The inhibitory effect of casticin on HepG2 and MCF-7 and the cellular uptake mechanism might provide preliminary directions for cancer treatment. The anti-cancer activity of the blank emulsion, free casticin, and casticin-loaded nanoemulsion on MCF-7 and HepG2 cells was measured by the MTT test. The relationship between the dosage of casticin and cell viability is shown in Figure 4. The blank nanoemulsion showed very low cytotoxicity. The cell survival rate in MCF-7 cells was about 90% and was almost 100% in HepG2 cells. Therefore, the whey protein isolate-lactose conjugate-stabilized nanoemulsion could be considered a safe drug-delivery vehicle. Over 24 h, as the concentration of free casticin increased to 100 μg/mL, there was a significant inhibitory effect on MCF-7 cells, whereas 50 μg/mL free casticin significantly inhibited the HepG2 cells. Compared with free casticin, when the concentration of casticin in the nanoemulsion reached 25 μg/mL, the inhibitory effect on MCF-7 and HepG2 cells was obvious. After 48 h, the inhibitory effect of the casticin-loaded nanoemulsion on cells was significantly higher than that of free casticin. The casticin-loaded nanoemulsion has a nanometer particle size, which allows it to enter cells through endocytosis and release casticin in the endosomes and lysosomes over a long period of time. The controlled-release of bioactive compounds from a nano-carrier can greatly improve the efficacy of the encapsulated substance [25].

### 3.5. Cellular Uptake of the Casticin-Loaded Nanoemulsions

**Figure 5:**
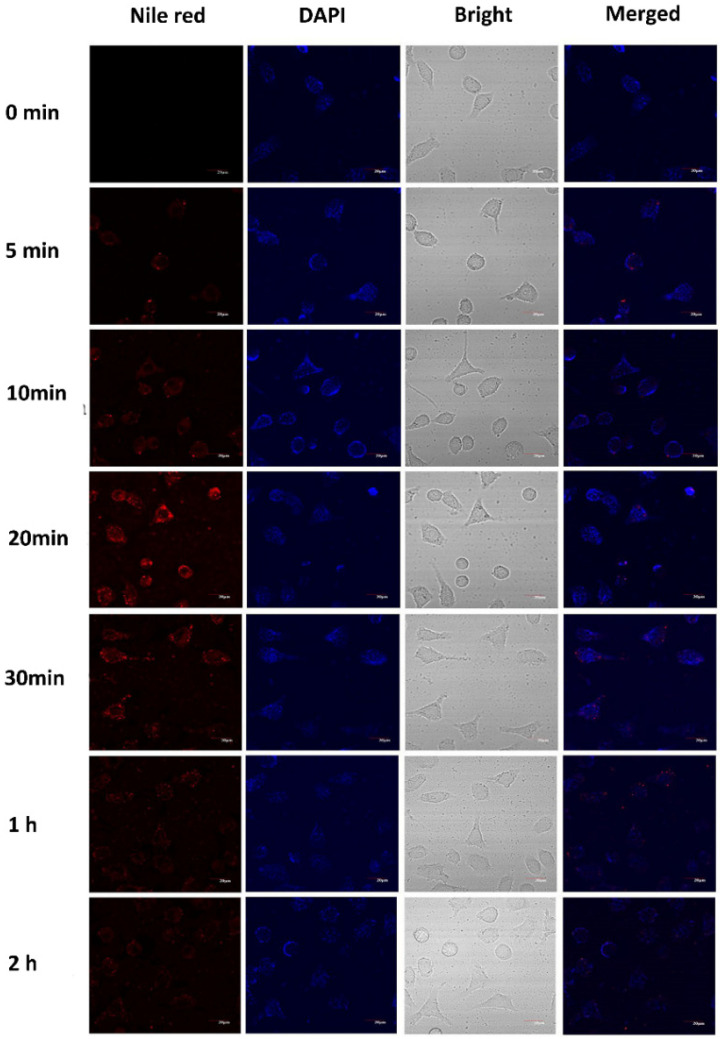
Cellular uptake observed by CLSM of cultured MCF-7 after treatment with CAS-loaded nanoemulsions. Cell nuclei were stained with DAPI for visualization in blue, and nanoemulsions were dyed with Nile red in red.

**Figure 6:**
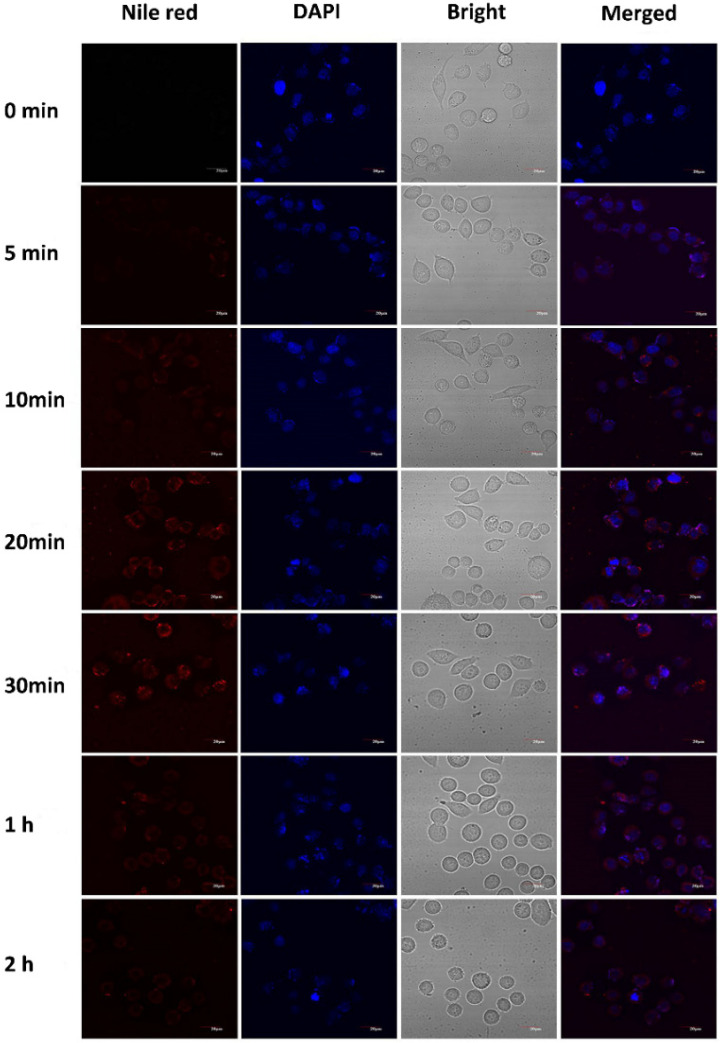
Cellular uptake observed by CLSM of cultured HepG2 after treatment with CAS-loaded nanoemulsions. Cell nuclei were stained with DAPI for visualization in blue, and nanoemulsions were dyed with Nile red in red.

The uptake of the casticin-loaded nanoemulsion in the MCF-7 and HepG2 cells observed by CLSM is shown in Figure 5 and Figure 6, respectively. The casticin-loaded nanoemulsion was labeled with Nile red, and the nuclei were stained with DAPI. After culturing the MCF-7 cells with the casticin-loaded nanoemulsion, a small amount of red fluorescence appeared dispersed in the lysosome of the cells after 5 min. The intensity of the red fluorescence in the cells was significantly enhanced after 20 min, which meant that the MCF-7 cells were continuously taking up the casticin-loaded nanoemulsion in a time-dependent manner. The red fluorescence in the MCF-7 cells gradually became weak and disappeared completely after 2 h. Peng et al. found that nanoparticles were easily absorbed by cells if their size was less than 200 nm [26]. Because the average particle size of the casticin-loaded nanoemulsion was 180 nm, one possible reason for the gradual disappearance of fluorescence could be related to the decomposition of the casticin-loaded nanoemulsion over a prolonged period of time. Additionally, the areas of red and blue fluorescence did not overlap, suggesting that the casticin-loaded nanoemulsion did not enter the nucleus but stayed in the cytoplasm. The cellular uptake phenomenon in the HepG2 cells was similar to that of the MCF-7 cells, but the optimized uptake time was 30 min. 

**Figure 7:**
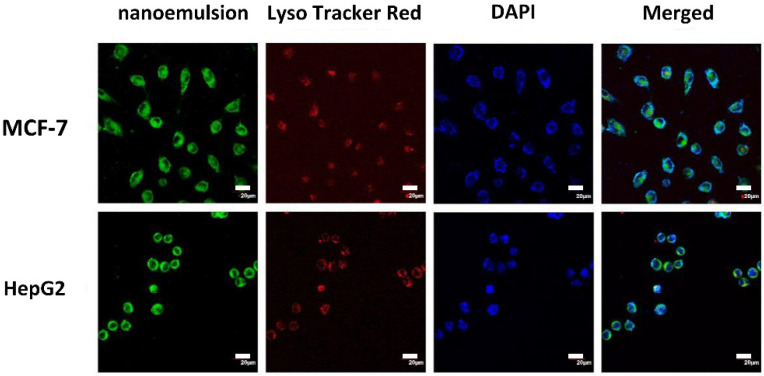
Cellular localization of CAS-loaded nanoemulsions after incubating with MCF-7 and HepG2 for 20 min. Nanoemulsions, lysosomes, and nuclei were labeled by coumarin-6 (first column, green color), Lyso-Tracker red (second column, red color), and DAPI (third column, blue color), respectively. The scale bar in the CLSM image is 20 μm.

To clarify the mechanism of the inhibition of cancer cells by the casticin-loaded nanoemulsion, the determination of cell localization was carried out. The casticin-loaded nanoemulsion was labeled with coumarin-6 (green color); the cell nuclei were dyed with DAPI (blue color), and the lysosomes were labeled with LysoTracker red. As shown in Figure 7, the casticin-loaded nanoemulsion was distributed in the cell cytoplasm after entering the cell. The merged view shows that the red fluorescence of the lysosome is overlapped by the green fluorescence of the casticin-loaded nanoemulsion, while the blue fluorescence of the nucleus is distinct. This indicated that the casticin-loaded nanoemulsion entered the lysosome after cellular uptake but did not enter the nucleus. Many studies have shown that nanomaterials enter cells through endocytosis to form endosomes and then are accumulated in lysosomes [27]. Kam et al. labeled single-walled carbon nanotubes (SWNT) and co-cultured them with HL60 cells. They found that SWNTs were distributed in the cytoplasm, and mainly concentrated in the lysosomes rather than entering the nucleus [28]. The cell recognized the nanomaterials as alien invaders and then transported them to the lysosome for degradation [29].

### 3.6. Cellular Uptake Mechanism

Cellular uptake is the process by which cells absorb molecules or materials from the extracellular space. Nanoparticles are taken into cells mainly through endocytosis [30,31], which is divided into phagocytosis and pinocytosis. Phagocytosis mainly occurs in phagocytic cells, such as fibroblasts and epithelial cells [32]. It is usually used for explaining the uptake of particles larger than 750 nm by mammalian cells [33,34]. Based on the protein-induced pathway, pinocytosis is subdivided into clathrin-dependent, caveolae-dependent, and macro-pinocytosis [35].

**Figure 8:**
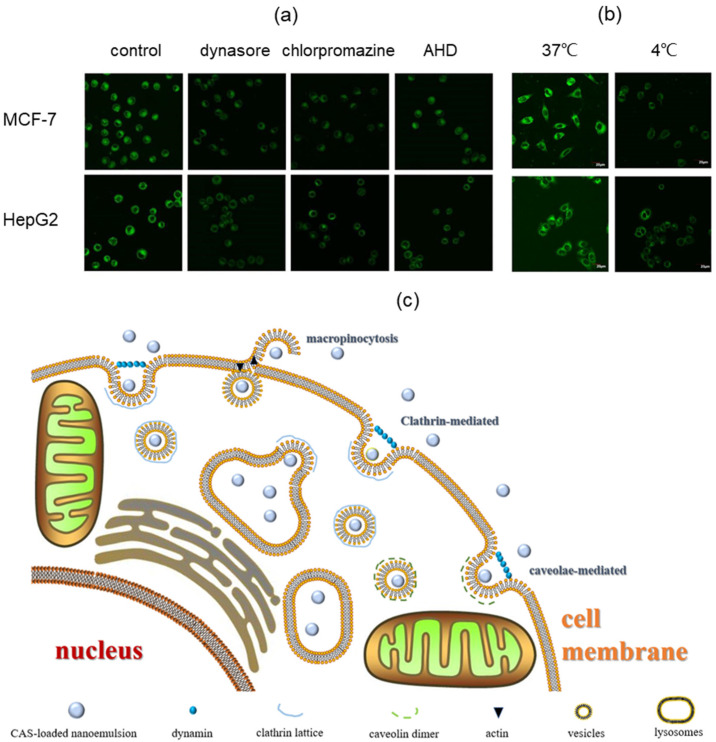
(**a**) Cellular uptakes of CAS-loaded nanoemulsions after incubating with MCF-7 and HepG2 after treatment with specific endocytosis inhibitors; (**b**) cellular uptake of CAS-loaded nanoemulsions after incubating with MCF-7 and HepG2 under different temperatures (37 °C and 4 °C) treatment for 30 min. The control was incubated with nanoemulsions without inhibitors. (**c**) Illustration of the protentional endocytic pathways for uptake of CAS-loaded nanoemulsions.

Herein, three pathway inhibitors were used to investigate some of the specific pathways involved during the cellular uptake of the casticin-loaded nanoemulsion. Dynasore inhibits dynein-dependent cellular uptake, such as clathrin-mediated and caveolae-mediated pits [36]. Chlorpromazine inhibits the transport-related molecule, resulting in the inhibition of the clathrin-mediated pathway [37]. Amiloride HCl dihydrate reduces the pH of the membrane surface to block Rac1/Cdc42 signal transmission, which induces a decrease of micropinocytosis [38]. In Figure 8a, as for MCF-7 cells, dynasore and chlorpromazine were more potent inhibitors of the casticin-loaded nanoemulsion than amiloride HCl dihydrate. This suggested that a clathrin-mediated pit might be the main method for the uptake of the casticin-loaded nanoemulsion into MCF-7 cells, and this might be accompanied by a small amount of macro-pinocytosis.

As for HepG2 cells, the dynasore and amiloride HCl dihydrate inhibitors had more influence on the cellular uptake of the casticin-loaded nanoemulsion, but chlorpromazine showed limited inhibition. Therefore, macro-pinocytosis is likely the main pathway for the entry of the casticin-loaded nanoemulsion, as well as the involvement of a small amount of clathrin-mediated and caveolae-dependent pits. The results showed a little bit of similarity to silver nanoparticles [39] and silica nanoparticles [40]. 

Temperature affects the entry of nanoemulsions into cells. At a low temperature, the fluidity of the cell membrane become weak, and the activity of enzymes involved in uptake activities is significantly reduced, resulting in a decrease in energy-dependent cellular uptake. As shown in Figure 8b, the MCF-7 and HepG2 cells were incubated with the casticin-loaded nanoemulsion at 37 °C and 4 °C, respectively. The low-temperature pretreatment significantly reduced the uptake of the nanoemulsion in these two cell lines. We speculated that the uptake of the casticin-loaded nanoemulsion required processes that use energy, including macro-pinocytosis, phagocytosis, and clathrin-mediated endocytosis or the use of a caveolae-dependent pit.

Figure 8c summarizes a partial schematic diagram of the casticin-loaded nanoemulsion entering cells through endocytosis and reaching the lysosomes. In short, there are three ways for the casticin-loaded nanoemulsion to enter the cell. The first is clathrin-mediated endocytosis. The casticin-loaded nanoemulsion reaches the cell surface, and vesicles are formed under the action of dynamin and the clathrin lattice to provide for the entry of the nanoemulsion into the cell; after that, it combines with lysosomes for degradation. The second way is through micropinocytosis. Under the action of actin, the surface of the cell membrane folds and stretches to form a groove; the nanoemulsion enters the groove during the stretching of the cell membrane and then enters the cell membrane. The third way is through the small part of a caveolae-mediated pit. Under the mediation of a dynamin and caveolin dimer, the cell membrane depression allows the casticin-loaded nanoemulsion to enter the cell. Finally, the main pathway for nanoemulsion to enter HepG2 cells is macro-pinocytosis, through which it entered MCF-7 predominantly through the clathrin-mediated pit.

## 4. Conclusions

The nanoemulsion prepared by MWPI has better stability than that prepared by WPI. The casticin-loaded nanoemulsion was fabricated by a MWPI stabilized with MCT. The loading amount of casticin in the nanoemulsion after spray-drying reached 700 μg/g. The casticin-loaded nanoemulsion had good physical stability in the DMEM medium as its particle size was maintained at 200 nm over 8 h with a small PDI (<0.2). Compared with free casticin, the casticin-loaded nanoemulsion showed a stronger inhibitory effect on MCF-7 and HepG2 cells. The inhibitory effect was dependent on the dosage of casticin and the co-culturing time. The phagocytosis of the casticin-loaded nanoemulsion by MCF-7 and HepG2 cells was time-dependent. Over time, the cellular uptake of the casticin-loaded nanoemulsion increased. The casticin-loaded nanoemulsion entered the cell by co-localization with the intracellular lysosome, indicating that a portion of the casticin-loaded nanoemulsion entered the cell via the lysosome. The main mechanism for the casticin-loaded nanoemulsion to enter the cells was via pinocytosis for HepG2 and clathrin-mediated pits for MCF-7 cells.
